# PD-L1 expression and tumor mutational burden status for prediction of response to chemotherapy and targeted therapy in non-small cell lung cancer

**DOI:** 10.1186/s13046-019-1192-1

**Published:** 2019-05-14

**Authors:** Yanhui Chen, Quanxing Liu, Zhiming Chen, Yating Wang, Wanning Yang, Ying Hu, Wenbo Han, Hui Zeng, Haitao Ma, Jigang Dai, Henghui Zhang

**Affiliations:** 1 Institute of Infectious Diseases, Beijing Ditan Hospital, Capital Medical University, Beijing Key Laboratory of Emerging Infectious Diseases, No.8 Jingshundongjie, Beijing, 100015 China; 2 Department of Thoracic Surgery, Xinqiao Hospital, Third Military Medical University (Army Medical University), 183 Xin Qiao Zheng jie, Chongqing, 400037 China; 3 Department of Thoracic Surgery, Huashan Hospital, 12 Wu Lu Mu Qi Road (M), Shanghai, 200040 China; 4 Genecast Precision Medicine Technology Institute, Huayuanbeilu 35, Beijing, 100089 China; 5 Department of Thoracic Surgery, The First Affiliated Hospital of Soochow University, No. 188 Shizi Street, Suzhou, 215006 China

**Keywords:** PD-L1, TMB, Biomarker, Targeted therapy, Non-small cell lung cancer, Prognosis

## Abstract

**Background:**

Several targeted immunotherapies have recently showed significant advances in treatment of non-small cell lung cancer (NSCLC), including antibodies and inhibitors targeting programmed death-1 (PD-1) and its ligand (PD-L1).

**Methods:**

Tumor tissue samples were prospectively collected from 183 patients with NSCLC including lung adenocarcinoma (ADC) and squamous cell carcinoma (SQCC). PD-L1 expression level was measured by immunohistochemistry assay and tumor mutational burden (TMB) status was assessed by next generation sequencing. Correlations between PD-L1 expressions, TMB status with clinicopathological characteristics were analyzed.

**Results:**

PD-L1 expression was detected in 37% of ADC group and 55% in SQCC group while all clinicopathological characteristics were found comparable between these two groups. PD-L1 expression was negatively associated with overall survival in ADC group (*P* < 0.0001) but not in SQCC group (*P* = 0.418). In consistent with PD-L1 expression level, TMB status was significantly lower in ADC subjects as compared to SQCC subjects (*P* = 0.024) while PD-L1 positive subgroup and TMB high subgroup shared less subjects within ADC group than SQCC group. More importantly, the combination of TMB status and PD-L1 expression successfully identified responders, who showed significant longer median overall survival than non-responders (32 months vs. 8.5 months) in ADC subjects (*P* < 0.0001) but not in SQCC subjects.

**Conclusions:**

Here we tested the hypothesis that monitoring TMB, in addition to the existing PD-L1 expression level, could represent valuable non-invasive biomarkers for the chemotherapy and targeted therapy. Further analyses are in need to further assess the prognostic value of TMB for ADC and SQCC patients receiving immunotherapy**.**

**Electronic supplementary material:**

The online version of this article (10.1186/s13046-019-1192-1) contains supplementary material, which is available to authorized users.

## Background

Lung cancer is one of the most common malignancy and a leading cause of cancer death in the world [1, 2]. The last decade has witnessed remarkable progress in the development of checkpoint blockade immunotherapy, particularly drugs targeting programmed cell death 1 (PD-1) and programmed cell death ligand 1 (PD-L1) in non-small cell lung cancer (NSCLC) [3]. Antibodies and inhibitors targeting PD-1/PD-L1 have been approved for the treatment of patients with advanced/metastatic NSCLC not responding to platinum-based chemotherapy [4, 5].

Biomarkers predicting response to the immunotherapies allow early selection of responders and timely implementation of treatment options. Currently, patient selection is majorly based on PD-L1 expression level in tumor tissue while it has been noticed that PD-L1 testing alone is insufficient for patient selection. Low PD-L1 expression detected from squamous NSCLC (< 10%) did not successfully predict the response [6]. A number of studies have suggested that PD-L1 expression correlates with an increased response to therapies in NSCLC [7, 8]. However, this conclusion was recently challenged by a few independent studies because it was proved that PD-L1 expression as well as its prognostic value is dynamic and affected by methodology and selection of antibody [9]. Moreover, it is reported a certain amount of PD-L1-negative patients also respond to PD-1/PD-L1 inhibitors in spite of the high tumor heterogeneity [10, 11]. Therefore, PDL1 expression level alone is not considered a predictive biomarker of response, rather a risk factor useful to identify the patient who more likely will benefit from the therapy [12]. The development of new predictive biomarkers as well as validation of the associated clinical management decisions is a priority for checkpoint inhibitor-based immunotherapy.

In addition to PD-L1 expression levels in cancer cells, several candidate predictive biomarkers were investigated including gene alterations and phenotypic alternations [13, 14], tumor microenvironments and immune effector cells [15, 16], and clinicopathologic factors [17, 18]. Tumor mutational burden (TMB), defined as the total number of mutations per coding area of a tumor genome, is highly feasible nowadays in tumor samples and has emerged as a potential biomarker in cancer immunotherapy [19, 20]. Higher TMB significantly predicts favorable outcome to PD-1/PD-L1 blockade in both NSCLC and small-cell lung cancer, suggesting comprehensive genomic profiling may result in patient benefit [21, 22]. It remains unclear that whether TMB status is correlated with the prognosis of NSCLC patients to the traditional treatments, and what is the prognostic power of the combination of TMB with other biomarkers.

Here, we aim to determine whether TMB status, and/or in combination with PD-L1 expression, correlates with the prognosis in NSCLC patients. To this end, we performed a retrospective study correlating the presence of TMB and PD-L1 expression with patient survival as well as other clinicopathologic parameters for patients with NSCLC including lung adenocarcinoma (ADC) and squamous cell carcinoma (SQCC). We also compared patients with high TMB value with patients with positive PD-L1 expression and investigated whether there is some overlap between patient groups stratified based on the distribution of PD-L1 expression and TMB status.

## Material and methods

### Patient and clinical data

**Fig. 1:**
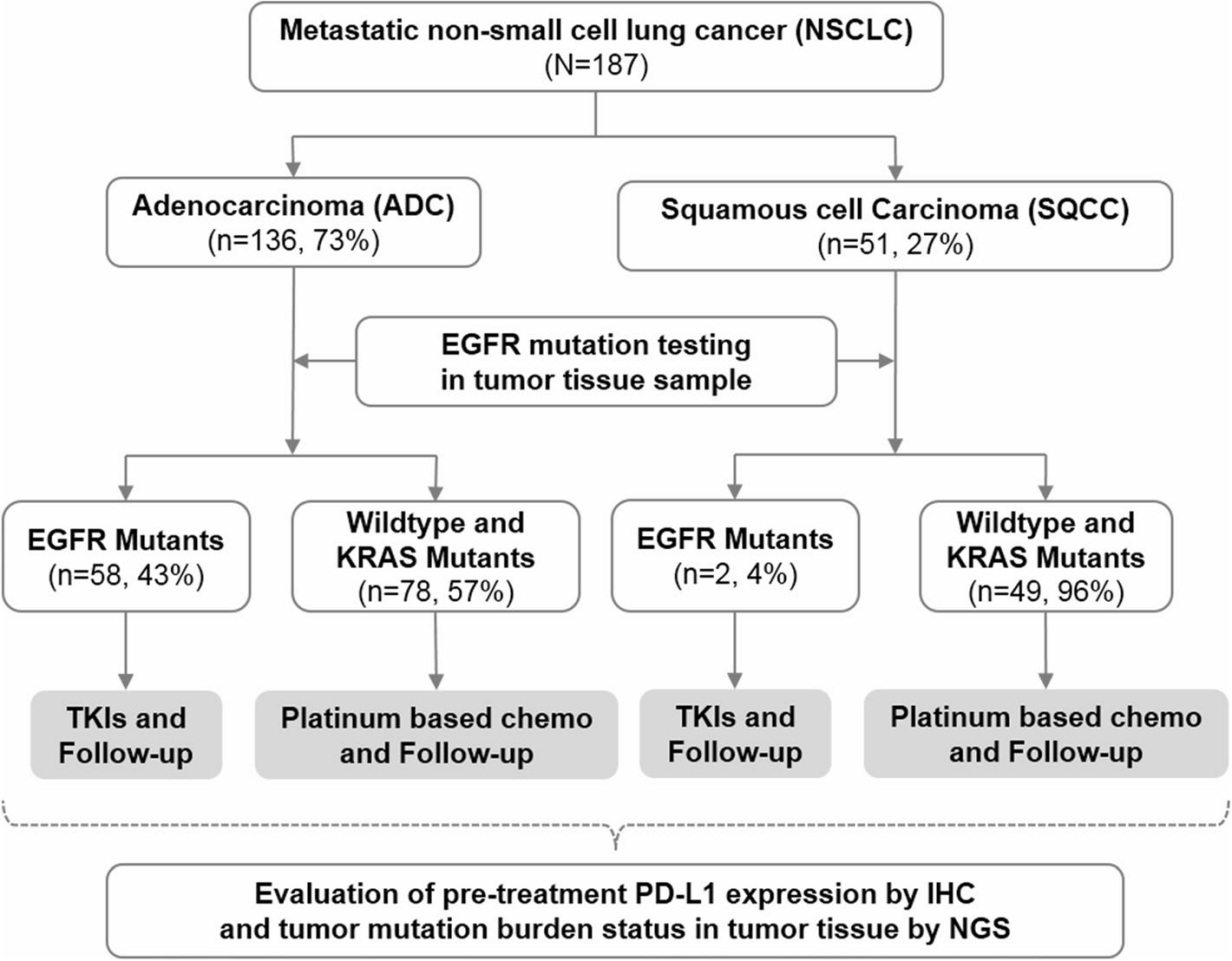
Workflow diagram. TKIs: tyrosine kinase inhibitors; Platinum-based chemo: platinum-based chemotherapy; PD-L1: programmed cell death-ligand 1; IHC: Immunohistochemistry; NGS: Next-Generation Sequencing

The study population consisted of 187 metastatic NSCLC patients who had received treatment at the Huashan Hospital, Third Military Medical University (Army Medical University), The First Affiliated Hospital of Soochow University and Beijing Ditan Hospital from November 2009 to July 2016. The patients were further analyzed if have sufficient paraffin-embedded tumor tissue for IHC staining to measure PD-L1 expression level and NGS sequencing to identify TMB biomarker status (Fig. 1). Patients with a prior history of malignant tumors or diagnosed with non-lung adenocarcinoma or non-lung squamous cell carcinoma, or with ALK, BRAF, ERBB2, MET, RET, or ROS1 mutations were excluded. Patients with EGFR mutations received EGFR-TKIs, while the other patients (e.g. KRAS mutations) received up to 6 cycles of platinum-based chemotherapy (docetaxel combined with cisplatin, or carboplatin).

Clinical and pathological data, including sex, age at diagnosis, smoking history, tumor histology, pathologic stage, and mutation status, was collected in accordance with study protocol requirements (Table 1). The survival outcome data were observed at follow-up. Written consent was taken from all patients prior to the initiation of any study-related procedure, and the study was approved by the Ethics Committee of Beijing Ditan Hospital.

**Table 1:** Patient characteristics by study group

Characteristic	ADC (*n* = 136)		SQCC (*n* = 51)		*P* value
Age (years)					0.315
Median	58		60		
Range	29–82		30–80		
Sex					0.055
Male	86	63%	40	78%	
Female	50	37%	11	22%	
Smoking status					0.172
Never smoker	109	80%	36	71%	
Smoker	27	20%	15	29%	
Pathologic stage					0.868
IIIB	54	40%	21	41%	
IV	82	60%	30	59%	
Mutation status					<0.0001
EGFR mutant	58	43%	2	4%	
KRAS mutant	17	12%	2	4%	
Other	61	45%	47	92%	

### Gene mutation screening

All patients were screened for the presence of gene mutations. Tumor DNA and RNA was co-extracted from each tissue specimen according to standard protocols (RNeasy Mini Kit, and QiAa-mp DNA Mini Kit, Qiagen, Hilden, Germany). Total RNA samples were reverse transcribed into single-stranded cDNA using a RevertAid First Strand cDNA Synthesis Kit (Fermentas, St. Leon-Rot, Germany). Either genomic DNA or cDNA was used for polymerase chain reaction (PCR) amplification. Selected sequencing of various genes, including EGFR (exons 18 to 22), ERBB2 (exons 18 to 21), KRAS (exons 2 to 3), and BRAF (exons 11 to 15), were screened (direct PCR amplification using cDNA) and/or further sequenced to precisely identify the mutations. FISH assays and real-time PCR were simultaneously performed to detect ALK, ROS1, MET and RET translocations.

### Next-generation sequencing (NGS)

DNA was isolated from blood sample for DNA sequencing analysis. A total of 2 ml of whole blood was collected from each patient, and the peripheral blood lymphocytes was isolated for DNA extraction using the Tiangen Whole Blood DNA Kit (Tiangen, Beijing, PRC) according to the manufacturer’s instructions. DNA concentration was measured and normalized using the Qubit dsDNA HS Assay Kit or Qubit dsDNA BR Assay Kit (Life Technologies, CA, USA). Genomic DNA was sheared into 150–200-bp fragments with Covaris M220 Focused-ultrasonicatorTM Instrument (Covaris, MA, USA). Fragmented DNA libraries were constructed by a KAPA HTP Library Preparation Kit (Illumina platforms) (KAPA Biosystems, MA, USA) following the manufacturer’s instruction. DNA libraries were sequenced by a custom-designed assay that comprised a hybridization capture-based Genescope panel of 1086 genes (Genecast, Beijing, China). This sequencing was mostly performed using ‘hotspot’ or targeted panels of known cancer-associated genes. This non-uniformity of coverage is mostly local (focused on a given exon) and partly global (focused on some exons across the genome). The paired-end sequencing was performed by Illumina HiSeq X-Ten. The hg19 reference genome was used for read mapping with BWA 0.7.12 (default parameters).

### Tumor mutational burden analysis

The TMB was defined as the number of somatic, coding, base substitutions, and indel mutations identified by NGS. All base substitutions and indels in the coding region of the targeted genes, including synonymous alterations, were initially counted before filtering as described above. Synonymous mutations were counted to reduce sampling noise, while non-coding alterations, germline alterations occurring with two or more counts in the ExAC database, alterations that were predicted to be germline by the somatic-germline zygosity algorithm, and any known germline alterations in dbSNP were excluded [23]. To calculate the TMB per megabase, the total number of mutations counted was divided by the size of the coding region of the targeted territory. The patients were stratified into 3 groups (high, moderate and low) according to the TMB level. Cut-off was selected to categorize patients into high and moderate groups (cut-off = median + SD), or moderate and low groups (cut-off = median-SD/2).

### Bioinformatics pipeline

Paired-end reads generated from the Hiseq X-Ten platform were sorted, filtered and indexed with SAM tools. To identify somatic SNP and indel mutations, the obtained BAM files from both tumor tissue samples and peripheral blood lymphocytes for each patient were processed for pairwise variant calling using VarScan (v2.4.2) [24] according to the following parameters. i) The minimum coverage for calling somatic variants in the peripheral blood lymphocyte samples was either 8×, or 6× for calling in tumor tissue samples; the *P* value threshold to call a somatic site was 0.05. ii) Variants with < 90% strand bias were kept for further study. The generated candidate mutations were annotated using Annovar software tools [25], and the dbNSFP and Exome Aggregation Consortum (ExAC) database was used to filter out either the benign mutations with pp2_hdiv score < 0.452 or the population polymorphic sites. Finally, the resulting nonsynonymous mutations at the exonic regions were kept. During the software working procedure, three main sources of bias that induce the extraneous variability of the sequencing read depth, which included the GC content, target footprint size and spacing, and the repetitive sequences, were also evaluated and corrected.

### Immunohistochemical (IHC) staining of PD-L1

The expression of PD-L1 on the surface of tumor cells (TC) and tumor-infiltrating immune cells (IC) was assessed through IHC staining. Paraffin-embedded tumor tissue was sectioned at a thickness of 4 μm and stained with a Ventana GX automated system (Ventana, AZ, USA). The tissue slides were stained by anti-PD-L1 (SP142) rabbit monoclonal primary antibody and a matched rabbit immunoglobulin G-negative control. The IHC signal was detected with the Ventana Amplification Kit and Ventana ultraView Universal DAB Detection Kit. Digital images were captured using Aperio Scanscope AT Turbo slide scanner under 20× magnification. Hematoxylin and eosin staining was also performed for all cases to orientate the pathologists’ reading.

Two pathologists, whom were both experts in interpreting the clinical cutoffs of the assays, independently evaluated all immunostained slides and there was no discrepancy review for discordant results. Scoring of PD-L1 expression intensity was performed using digital image analysis software (Aperio membrane v9 and Aperio Genie Classifier). The following analytical components were assessed based on standards reported in previous studies [5, 26, 27]. In brief, two scores were identified and evaluated by the morphologic features: 1) the TC score was defined as the percentage of PD-L1-expressing tumor cells (TC3, ≥50%; TC2, 5 to < 50%; TC1, ≥1 to < 5%; and TC0, < 1%); 2) the IC score was defined as the percentage of the tumor area (IC3, ≥10%; IC2, ≥5 to < 10%; IC1, ≥1 to < 5%; and IC0, < 1%). Together, a semiquantitative scoring estimation was used to calculate PD-L1 expression levels: TC0 and IC0 represent PD-L1 negative (−), TC1 or IC1 represent PD-L1 weak positive (+), TC2 or IC2 represent PD-L1 moderate positive (+), and TC3 or IC3 represent PD-L1 strong positive (+).

### Statistical analyses

Statistical analyses were performed using GraphPad Prism (version 7.01, La Jolla, CA, USA) and SPSS version 22.0 (SPSS, Inc., Chicago, IL, USA). Associations of PD-L1 expression, TMB status, and/or with clinicopathologic features were evaluated with Pearson’s Chi-squared test or Fisher’s exact test. Overall survival (OS) was defined as the time from the date that therapy started to the date of death from any cause or the date of last follow-up, with 95% confidence intervals calculated using the Kaplan-Meier method. Between-group comparisons in survival analysis were performed using the log rank test. The Kruskal-Wallis test was used to compare difference between multiple groups while the Dunn’s multiple comparisons test was used to compare difference between two groups. Spearman correlation analysis was performed to analyze the correlation of PD-L1 expression and TMB status. All tests were 2-sided and *P* < 0.05 was considered significant unless otherwise specified.

## Results

### ADC and SQCC patients share similar characteristics

A total of 187 NSCLC subjects composed of an ADC study group (*n* = 136, 73%) and a SQCC study group (*n* = 51, 27%) were enrolled in this retrospective study. There was no significant difference between ADC and SQCC groups in terms of age, sex, smoking status or pathological stage (Table 1). Interestingly, 58 (43%) ADC patients had EGFR gene mutation and 17 (12%) ADC patients had KRAS gene mutation, which are significantly higher than those in SQCC group (4% EGFR mutation and 4% KRAS mutation) (Table 1). Of all the EGFR mutation subjects, 33 subjects had L858R mutation and 25 subjects had exon 19 Del mutation. Of all the KRAS mutation subjects, there were 2 subjects with A146V mutation, 1 with A146X mutation, 1 with G12A mutation, 3 with G12C mutation, 2 with G12D mutation, 6 with G12 V mutation, 1 with G13D mutation, and 1 with Q61L mutation.

### PD-L1 expression is lower in ADC than SQCC

**Fig. 2:**
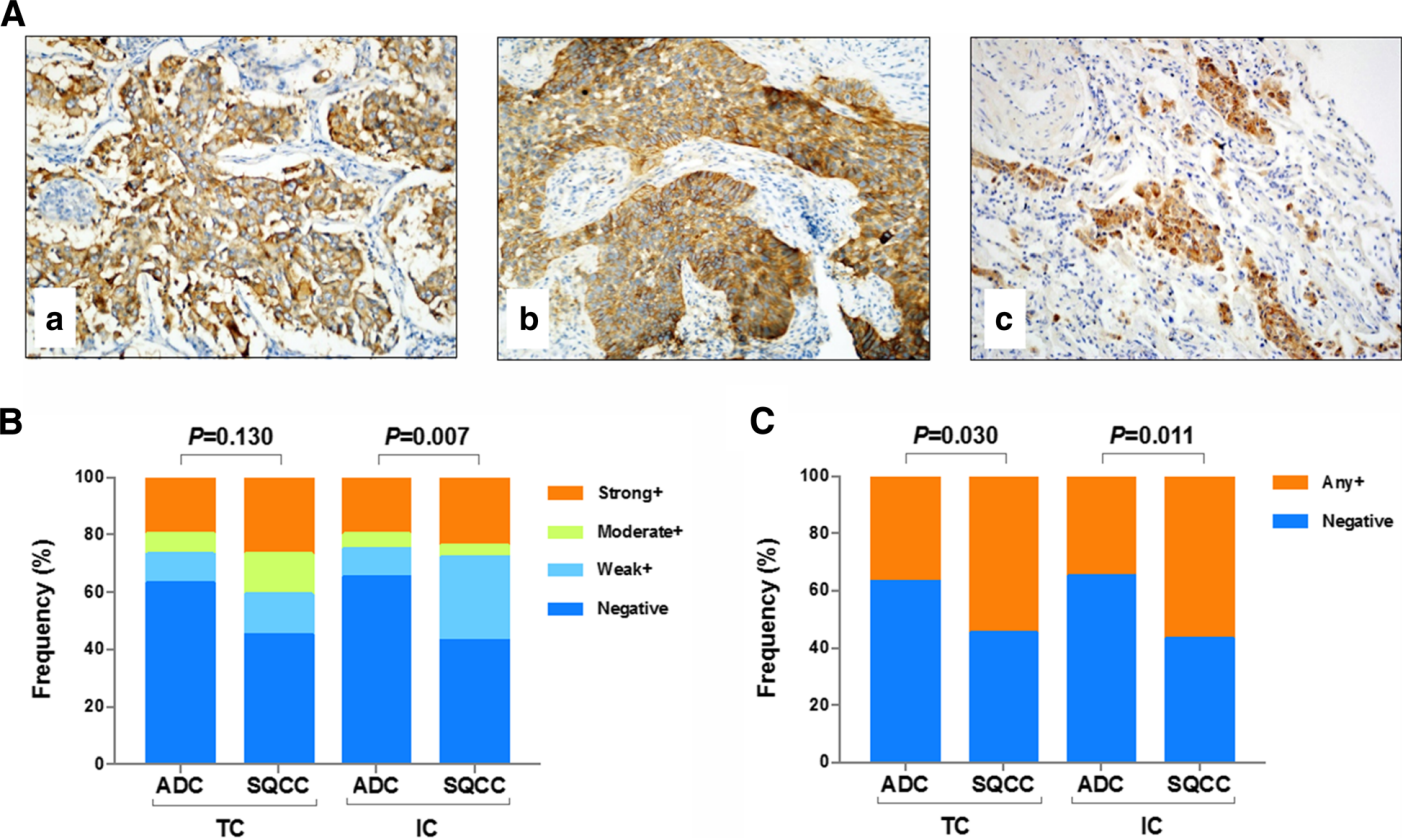
PD-L1 expression in ADC and SQCC study groups. Representative images of PD-L1 expression in cell membrane of tumor cells (TC) from ADC (**Aa**) and SQCC (**Ab**) subjects, and in cytoplasm from ADC subjects (**Ac**). Magnification, × 20. **B**: The percent frequencies of PD-L1 intensity in four categories (negative, weak, moderate and strong positive) in TC and immune cells (IC) from subjects as indicated. **C**: The percent frequencies of PD-L1 intensity in two categories (negative, and positive) as indicated

To investigate the expression pattern of PD-L1 in ADC and SQCC, PD-L1 protein level were evaluated in all 187 tumor tissue samples by IHC. Overall, 37% (any+) of ADC subjects and in 55% (any+) of SQCC subjects had tumors positive for PD-L1 expression. IHC analysis indicated PD-L1 protein located in cell membrane of tumor cells (Fig. 2Aa & Ab) or immune cells (data not shown) in most of ADC and SQCC samples. Of all 136 ADC patients, one patient was found with PD-L1 expressed in the cytoplasm (Fig. 2Ac). In ADC group, the percent frequencies of PD-L1 intensity in four categories (negative, weak, moderate and strong positive) were 63%/10%/7%/20% in TC and 65%/10%/5%/20% in IC, while the PD-L1 positivity was much higher in SQCC group (frequencies were 45%/14%/14%/27% in TC and 43%/29%/4%/24% in IC) (Fig. 2B). In consistent with that, comparison of PD-L1 intensity in two categories (negative and positive) showed significant higher expression in SQCC as compared to ADC (*P* = 0.030 in TC, *P* = 0.011 in IC) (Fig. 2C). Detailed comparison of PD-L1 expression in each subgroup stratified by demographics and clinical characteristics is showed in Table 2 and Table 3. Briefly, PD-L1 in TC is highly expressed in male ADC patients (*P* < 0.001) while in IC it is highly expressed in elder SQCC patients (> 60 yrs) (*P* = 0.011).

**Table 2:** Comparison of PD-L1 expression levels within ADC study group

Characteristic	PD-L1 expression									
	TC					IC				
	Negative (n, %)		Any+ (n, %)		*P* value	Negative (n, %)		Any+ (n, %)		*P* value
Age (years)					0.380					0.719
<58	43	67%	21	33%		40	63%	24	38%	
≥58	43	60%	29	40%		48	67%	24	33%	
Sex					<0.001					0.854
Male	45	52%	41	48%		55	64%	31	36%	
Female	41	82%	9	18%		33	66%	17	34%	
Smoking status					0.186					0.508
Never smoker	72	66%	37	34%		72	66%	37	34%	
Smoker	14	52%	13	48%		16	59%	11	41%	
Pathologic stage					0.586					0.098
IIIB	36	67%	18	33%		30	56%	24	44%	
IV	50	61%	32	39%		58	71%	24	29%	
Mutation status					0.049					0.475
EGFR mutant	43	74%	15	26%		38	66%	20	34%	
KRAS mutant	11	65%	6	35%		13	76%	4	24%	
Other	32	52%	29	48%		37	61%	24	39%

**Table 3:** Comparison of PD-L1 expression levels within SQCC study group

Characteristic	PD-L1 expression									
	TC					IC				
	Negative (n, %)		Any+ (n, %)		*P* value	Negative (n, %)		Any+ (n, %)		*P* value
Age (years)					0.579					0.011
<60	12	50%	12	50%		15	63%	9	38%	
≥60	11	41%	16	59%		7	26%	20	74%	
Sex					1					0.737
Male	18	45%	22	55%		18	45%	22	55%	
Female	5	45%	6	55%		4	36%	7	64%	
Smoking status					0.761					0.214
Never smoker	17	47%	19	53%		18	50%	18	50%	
Smoker	6	40%	9	60%		4	27%	11	73%	
Pathologic stage					1					0.579
IIIB	9	43%	12	57%		8	38%	13	62%	
IV	14	47%	16	53%		14	47%	16	53%	
Mutation status					\					\
EGFR mutant	1	50%	1	50%		1	50%	1	50%	
KRAS mutant	0	0%	2	100%		1	50%	1	50%	
Other	22	47%	25	53%		20	43%	27	57%	

### PD-L1 expression is negatively associated with overall survival in ADC group

**Fig. 3:**
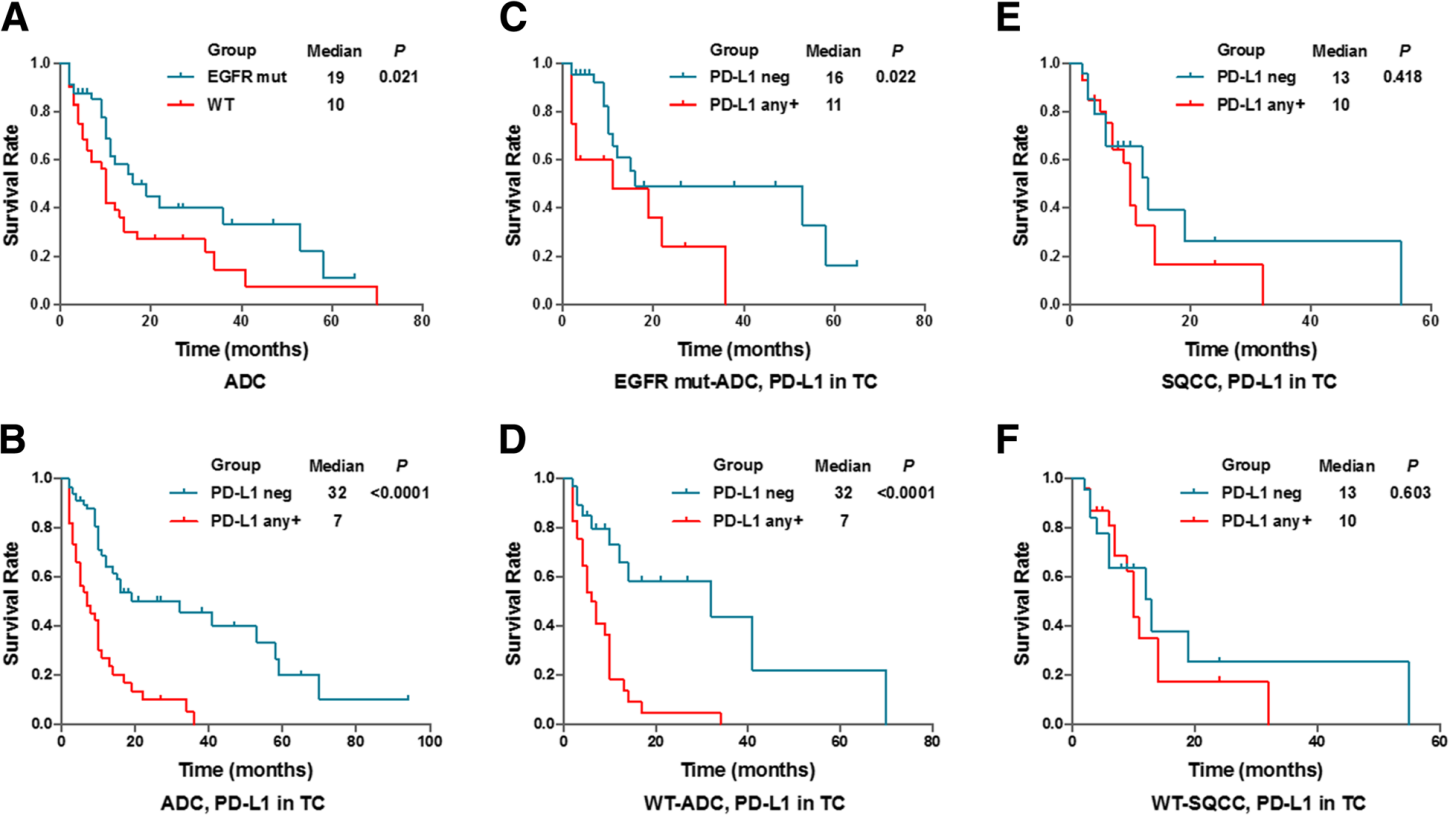
Survival analysis based on EGFR mutation and PD-L1 expression. **a**-**d**: Kaplan-Meier survival graph comparing ADC patients with wildtype (WT) and mutant (mut) EGFR (**a**), ADC patients with negative and positive PD-L1 expression (**b**), as well as comparing negative and positive PD-L1 subgroups in ADC patients either with mut EGFR (C) or WT EGFR (D) as indicated. **e**, **f**: Kaplan-Meier survival graph comparing PD-L1 negative and positive SQCC patients (**e**) and comparing PD-L1 negative and positive SQCC patients with WT EGFR (**f**)

The prognostic role of PD-L1 is not clear, consider PD-L1 expression was reported to associate with better prognosis, worse prognosis, or no prognostic significance. To explore if tumor PD-L1 expression is associated with prognosis in our study groups, Kaplan-Meier survival curve was generated to compare overall survival between various subgroups. For ADC subjects, the median overall survival was significantly longer in EGFR mutated group vs. wildtype group, as well as in PD-L1 (TC expression) negative group vs. positive group (*P* = 0.021 and < 0.0001, respectively) (Fig. 3a & b). Similar results were found in individual ADC subgroups with either mutated EGFR or wildtype (*P* = 0.022 and < 0.0001, respectively) (Fig. 3c & d). For SQCC subjects and wildtype SQCC subjects, no associations of tumor PD-L1 expression with prognosis was noticed between PD-L1 negative group vs. positive group (*P* = 0.418 and 0.603, respectively) (Fig. 3e & f). We did not observe significant association between PD-L1 expression in IC with prognosis in either ADC or SQCC group (data not shown), so only PD-L1 expression in TC is included in the following study. To investigate whether CD8 T cell responds differently between PD-L1 negative group vs. positive group, CD8 protein level in tumor-infiltration T-cells was stained and compared. As showed in the Additional file, comparison of the CD8 positive rate (Additional file 1: Figure S1A & S1B) or expression levels as distributed by quartiles (Additional file 1: Figure S1C & S1D) indicated there is no significant difference of CD8+ T cell infiltration between PD-L1 negative and PD-L1 any+ groups.

### TMB status may work as a potential biomarker for prediction

**Fig. 4:**
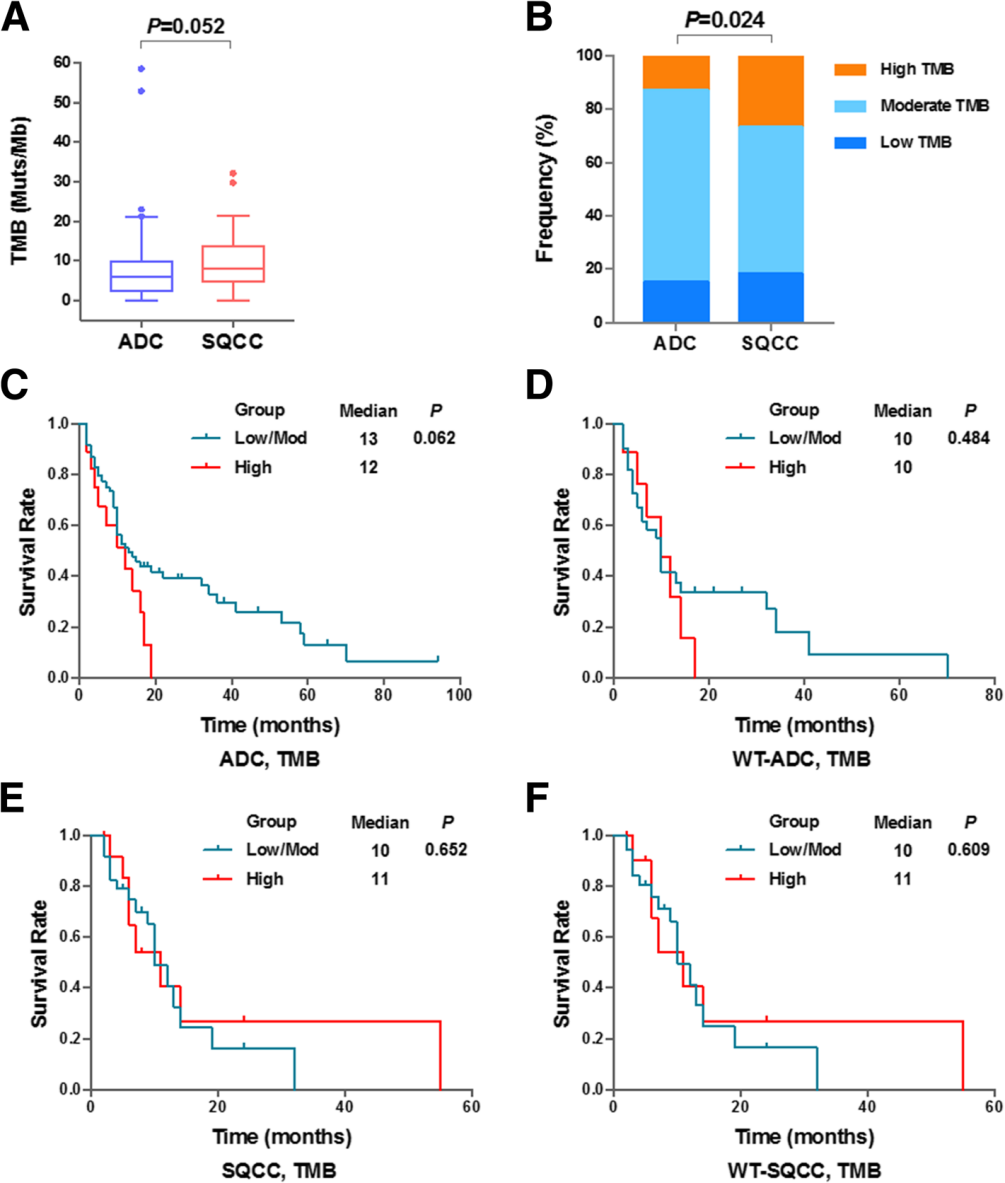
Characterization and survival analysis of Tumor Mutation Burden. **a**: Comparison of Tumor Mutation Burden (TMB) values in ADC and SQCC groups. **b**: The percent frequencies of TMB status in three categories (low, moderate and high) in two study groups. **c**-**f**: Kaplan-Meier survival graph comparing ADC patients with low or moderate (Low/Mod) TMB versus ADC patients with high TMB (**c**), WT-EGFR ADC patients with low/mod TMB and high TMB (**d**), SQCC patients with low/mod TMB and high TMB (**e**) and WT-SQCC patients with low/mod TMB and high TMB (**f**)

In this study, TMB was measured by a comprehensive genomic profiling (CGP) assay targeting 1086 cancer associated genes. The overall median TMB was 8.0 mutations/Mb, ranging from 0 to 58.5 mutations/Mb. In agree with high PD-L1 level detected in SQCC, the TMB value in SQCC group was found higher than ADC group although the difference was not significant (Fig. 4a). Next, the study subjects were divided into three groups based on TMB value: High (TMB ≥ 13.7), Moderate (2.2 ≤ TMB < 13.7) and Low (TMB < 2.2). Significant difference of TMB status was observed between two study groups (*P* = 0.024), suggesting lower TMB value in ADC patients which is in consistent with the lower PD-L1 expression detected in ADC patients (Fig. 4b).

It has been reported that higher TMB predicted favorable outcome to PD-1/PD-L1 blockade Immunotherapy in several cancers [20, 22, 28]. Study subjects were stratified into Low/moderate TMB group and High TMB group. As shown in Table 4, TMB status solely was not associated with the clinicopathological features with this cut-off setting. Moreover, Kaplan-Meier survival curve was generated to investigate the association between TMB statuses with patient prognosis. We have analyzed ADC (Fig. 4c) and SQCC subjects (4E), and subjects only with wildtype EGFR (Fig. 4d & f). Although no statistical significance of survival difference (all *P* > 0.05) was observed between Low/moderate TMB group and High TMB group, high TMB seem associate with poor prognosis in ADC but not in SQCC. Given TMB showed similar expression pattern with PD-L1 and related to poor survival events, we hypothesize TMB may form a biomarker signature set together with PD-L1.

**Table 4:** Comparison of TMB status in ADC and SQCC study groups

Characteristic	ADC				*P* value	SQCC				*P* value
	High (n, %)		Low/Moderate (n, %)			High (n, %)		Low/Moderate (n, %)		
Age (years)					0.805					1
<58 (< 60) #	9	14%	55	86%		7	29%	17	71%	
≥58 (≥60)	9	13%	63	87%		7	26%	20	74%	
Sex					0.068					0.704
Male	15	17%	71	83%		12	30%	28	70%	
Female	3	6%	47	94%		2	18%	9	82%	
Smoking status					1					0.512
Never smoker	15	14%	94	86%		11	31%	25	69%	
Smoker	3	11%	24	89%		3	20%	12	80%	
Pathologic stage					0.796					0.529
IIIB	8	15%	46	85%		7	33%	14	67%	
IV	10	12%	72	88%		7	23%	23	77%	
Mutation status					0.049					\
EGFR mutant	4	7%	54	93%		0	0%	2	100%	
KRAS mutant	5	29%	12	71%		2	100%	0	0%	
Other	9	15%	52	85%		12	26%	35	74%	
PD-L1 in TC										
Strong+	7	26%	20	74%	0.051	8	57%	6	43%	0.01
Moderate/strong+	8	22%	28	78%	0.084	10	48%	11	52%	0.01
Any+	9	18%	41	82%	0.293	11	39%	17	61%	0.058
Negative	9	10%	77	90%		3	13%	20	87%	
PD-L1 in IC										
Strong+	6	22%	21	78%	0.199	1	8%	11	92%	0.141
Moderate/strong+	6	18%	28	82%	0.389	1	7%	13	93%	0.076
Any+	8	17%	40	83%	0.431	7	24%	22	76%	0.752
Negative	10	11%	78	89%		7	32%	15	68%	

Note: #: for age of SQCC

### Correlations between PD-L1 (TC) and TMB and their overlaps in ADC and SQCC

**Fig. 5:**
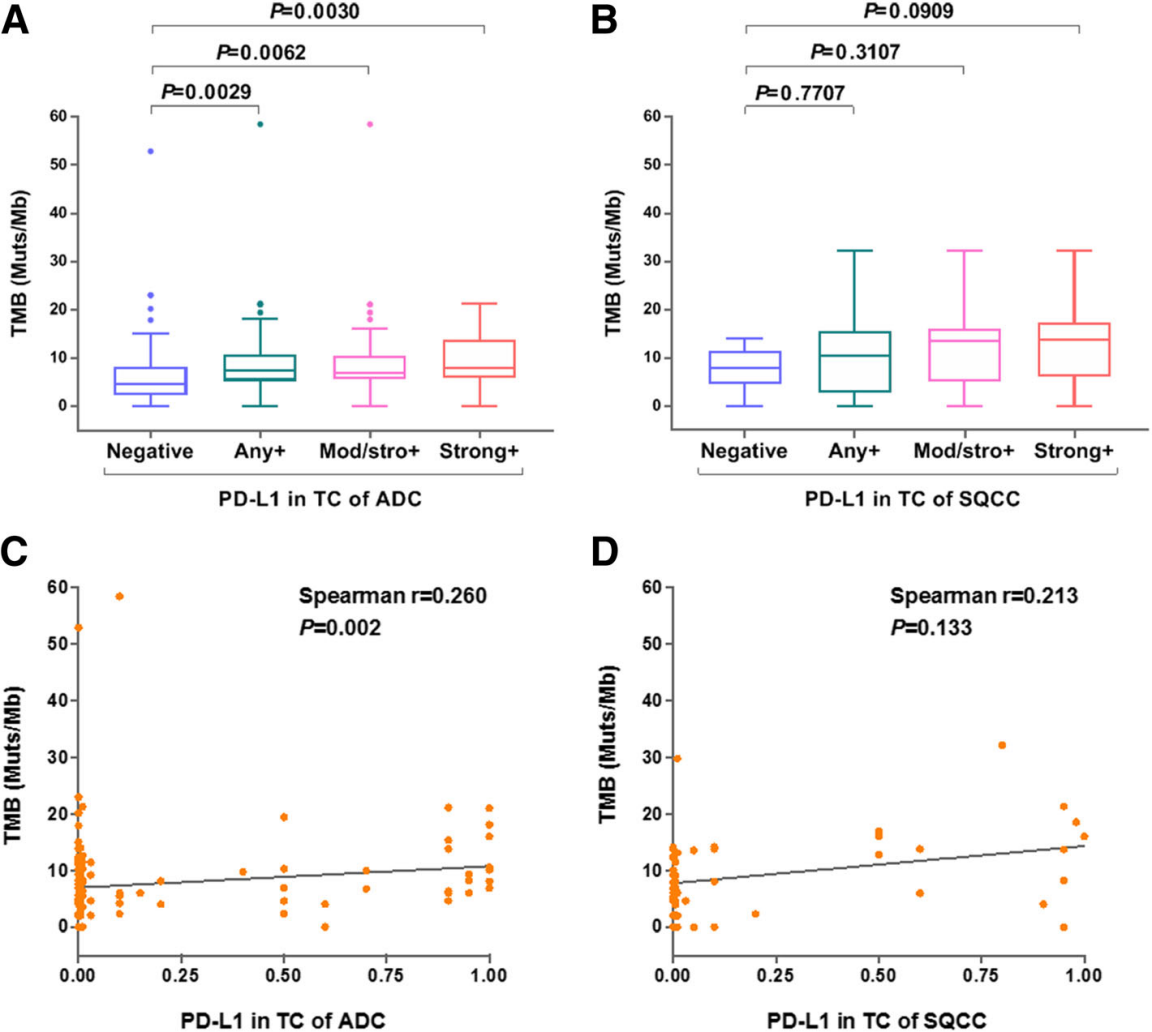
Association between PD-L1 (TC) expression and TMB status. **a**, **b**: Difference analysis of TMB values in ADC (**a**) and SQCC (**b**) subjects stratified by PD-L1 expression levels as indicated. **c**, **d**: Scatter plots and Spearman correlation coefficients between TMB values and tumor cell PD-L1 expression levels in ADC (**c**) and SQCC (**d**)

To test our hypothesis that TMB may act as complementary biomarker for PD-L1, we investigated the association between TMB values and PD-L1 positivity. In ADC group but not SQCC group, the TMB value of each PD-L1-positive subgroup (PD-L1+, or moderate/strong+, or strong+) was significantly higher than that of PD-L1 negative subgroup (*P* = 0.0029, *P* = 0.0062, *P* = 0.0030) (Fig. 5a & b). Spearman correlation analysis showed that PD-L1 expression and TMB value were not correlated either in ADC (Fig. 5c) or in SQCC (Fig. 5d).

**Fig. 6:**
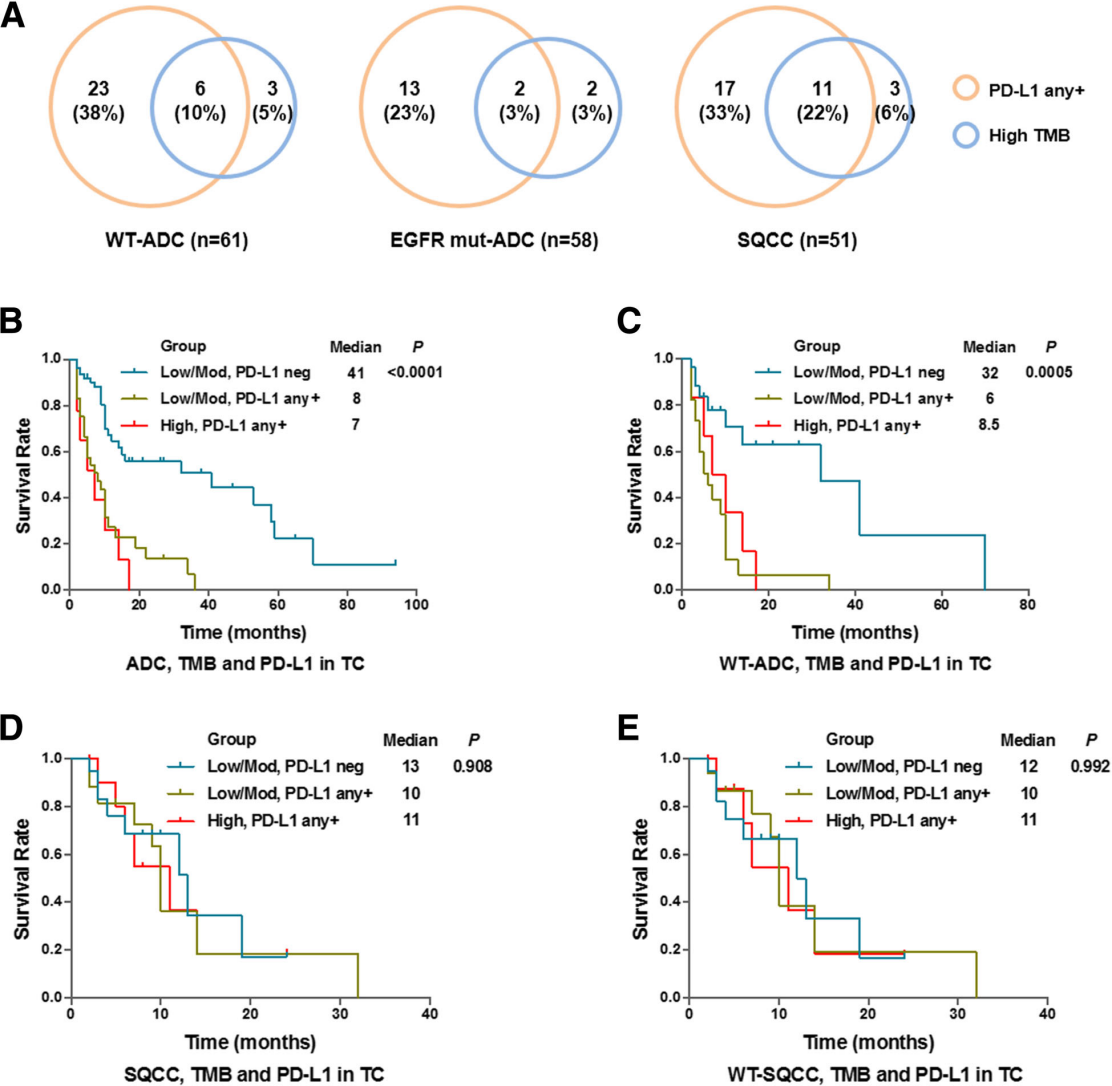
Combination analysis of PD-L1 expression and TMB status as a biomarker set. **a**: Venn diagram showing the overlap of PD-L1 positive and TMB high subjects in EGFR-WT ADC patients, EGFR-mut ADC patients, as well as SQCC patients. B-E: Kaplan-Meier survival curves comparing ADC subjects (**b**), EGFR-WT ADC subjects (**c**), SQCC subjects (**d**) and WT-SQCC subjects (**e**) as stratified based on both PD-L1 TC expression and TMB status as indicated

**Fig. 7:**
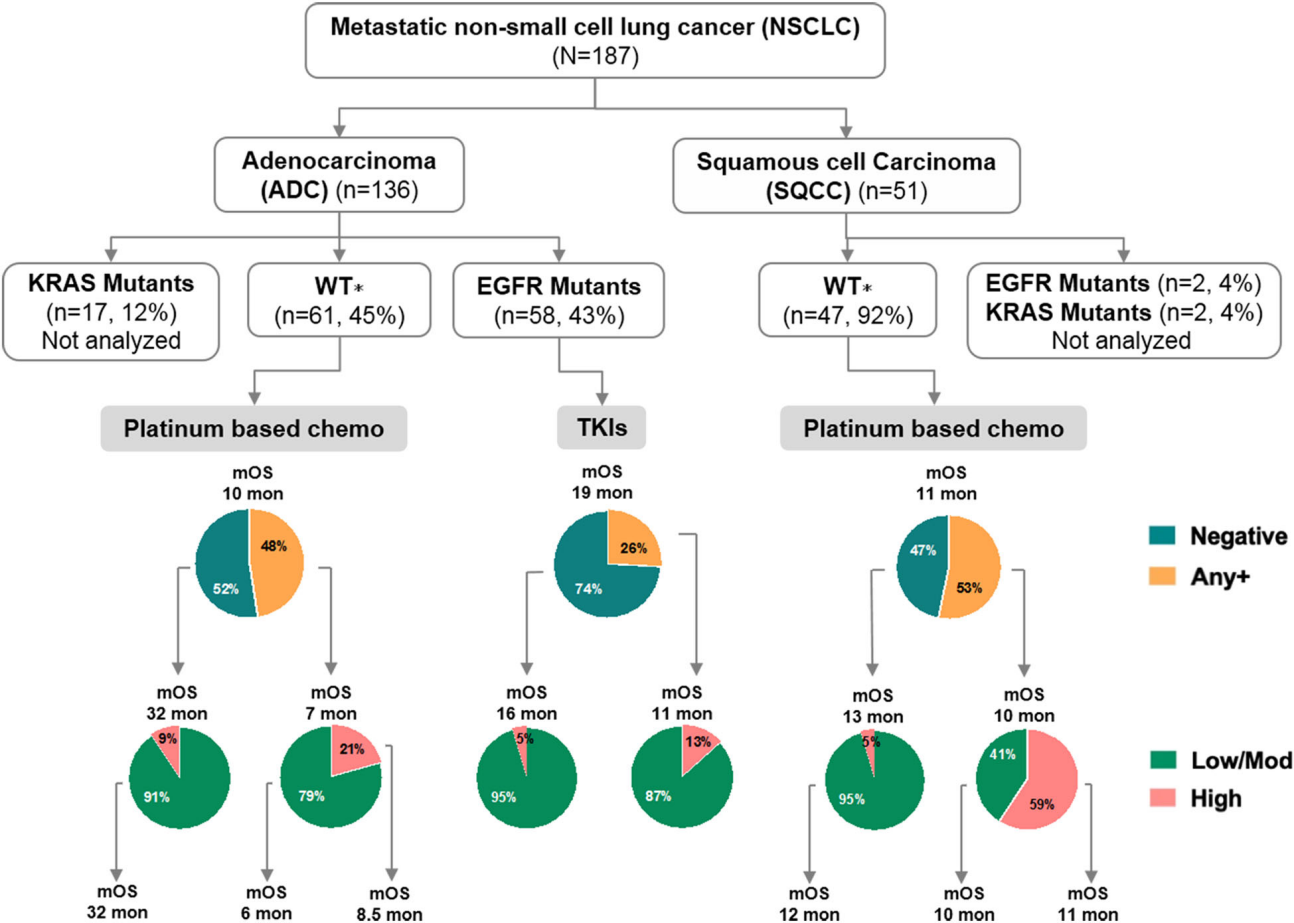
Summary of PD-L1 expression and TMB status in NSCLC. Teal blue and light orange pie chart indicate abundance of subjects grouped by PD-L1 positivity. Light green and orange red pie chart indicates abundance of subjects grouped by TMB status. mOS: median overall survival. ∗: Patients with both wildtype EGFR and KRAS

Next, we investigated the overlap of PD-L1 positive subjects and TMB high subjects in three study groups (Wildtype ADC, EGFR-mutant ADC and SQCC). There were only 10 and 3% patients of total is shared by PD-L1 positive subgroup and TMB high subgroup for ADC subjects, while much more patients (22%) is shared for SQCC subjects (Fig. 6a). This result, again, indicated the combination of TMB and PD-L1 as a biomarker set may exhibit better performance in predicting the outcome of patients. Hence, we combined cut off setting from both PD-L1 expression (TC) and TMB status and divided patients into three subgroups (low/moderate TMB + PD-L1 negative, low/moderate TMB + PD-L1 positive, and high TMB + PD-L1 positive). Not surprising, survival analysis showed the median survival in low/moderate TMB with negative PD-L1 subgroup is 41 months, which is remarkably longer than the other two subgroups in ADC subjects (Fig. 6b). Similar result was found in ADC subjects with wildtype EGFR subjects (Fig. 6c), but not in SQCC subjects or wildtype SQCC subjects (Fig. 6d & e). Given the wildtype ADC patients and SQCC patients received same treatment (platinum based chemotherapy), these results indicated TMB status combined with PD-L1 expression as a biomarker signature set allow identification of responders (medium survival = 32 months) and non-responders (medium survival = 6 or 8.5 months) specifically in ADC subjects but not in wildtype SQCC subjects (medium survival = 12, 10, or 11 months) (Fig. 7).

## Discussion

This retrospective study investigated PD-L1 expression as well as TMB status on the potential use of biomarkers in two NSCLC study groups. Our data showed that patients with ADC had higher PD-L1 expression and higher TMB value than SQCC although the no direct correlation of PD-L1 expression with TMB value was observed. Early survival analysis indicated negative association of PD-L1 expression with prognosis in ADC group but no significant association of TMB status with prognosis. Additionally, the combination of TMB status and PD-L1 expression successfully identified ADC responders with median overall survival at least 23.5 months longer with non-responders (32 months vs. 8.5 months), a difference that crossed the prespecified boundary for significance within other reported analysis. Therefore, this study confirmed the hypothesis that TMB could be used as a useful complementary biomarker with PD-L1 for ADC subjects.

In our study, the SP142 clone antibody was used to evaluate PD-L1 protein expression in tumor tissue samples. We detected positive TC staining of PD-L1 in 37% (any+) of ADC subjects and in 55% (any+) of SQCC subjects (Fig. 2). A comparable level of PD-L1 staining was observed in IC. This finding is in consistent with previous analysis in European and American cohorts [5, 26, 29]. Other studies reported lower PD-L1 positivity using antibody clone 28–8 (overall 31% in NSCLC, ≥1% cutoff) [30] and clone SP263 (overall 36.5% in NSCLC, ≥1% cutoff) [31]. The positive rates are even lower as measured by another PD-L1 antibody, clone 22C3 (4.4% any+ in ADC and 34.3% any+ in SQCC) [32]. The inconsistent results of PD-L1 positivity due to different clones was previously noticed [11]. Therefore, our study support that using PD-L1 expression as a biomarker of identifying therapy responder requires verification of detection and evaluation of consistency in multi-regional clinical centers. Several studies have reported that smoking history and/or gender was associated with better immunotherapy response [33–35]. In our study, higher PD-L1 expression was observed in male subjects (TC, ADC) as well as elder subjects (IC, SQCC) (Tables 2 & 3). This result is in agree with study reported by Chen et al. [32]. Our study also found that patients with ADC in our cohort have a higher positivity of EGFR mutations than the Western population, suggesting it is necessary for better management of targeted therapy for Asian population.

As more knowledge is gained about the predictive performance of PD-L1 in various human cancers, it seems that PD-L1 expression may show distinct characteristics in different cancers. PD-L1 expression was reported to be associated with poor prognosis in NSCLC [36, 37]. In our study, PD-L1 expression level was significantly lower in ADC as compared to SQCC (Fig. 2), and ADC subjects with negative PD-L1 expression had improved survival compared with the positive PD-L1 subgroup whereas no difference was noticed between these two groups in SQCC subjects (Fig. 3). When we preparing this manuscript, similar observations were reported by Korean scientists that PD-L1 expression is associated with shorter disease-free survival outcome but no associations in SQCC was noticed [38]. Therefore, negative PD-L1 expression may be used as an indicator of better survival specifically for ADC patients. This result is also in agreement with a progression-free survival analysis previously reported in a large cohort study [39]. In addition to that, as designed in this study, EGFR-TKIs were used as a standard treatment for EGFR-mutant patients included in this study. A longer survival was observed in EGFR-wildtype subjects (median survival = 32 months) compared to EGFR-mutant subjects (median survival = 16 months) who received chemotherapy, although in both groups PD-L1 negative subjects consistently showed significantly better survival (Fig. 3c & d). Together, these results suggested that PD-L1 expression may predict prognosis of ADC patients.

Recent evidences have shown that TMB status may act as a predictor for the efficacy of NSCLC PD-1/PD-L1 blockade therapy [19, 40]. Clinical trials found that the response rate to PD-1/PD-L1 inhibitors in high-TMB patients is substantially higher than in low-TMB patients [21, 41]. These results demonstrated that biomarkers like gene alteration information directly derived from tumor tissues are clinically relevant for immunological checkpoint inhibitor therapy. Our study found that the TMB value is higher in SQCC subjects rather than ADC subjects, which is in line with previous reports [42] (Fig. 4a & b). However, TMB solely does not significantly correlate with patient survival in our study (Fig. 4c-f).

Cancer patients with higher TMB was been hypothesized to carry more neoantigens that could be recognized by the immune system and lead to response to checkpoint inhibition. An important finding presented in this study is TMB value was significantly higher in PD-L1 positive subjects than PD-L1 negative subjects, indicating an interesting association between these two biomarkers (Fig. 5a). In our study, there were certain overlaps between high TMB and PD-L1 subjects in SQCC and minor overlaps between wild-type ADC and EGFR-mutant subjects (Fig. 6a). Similar results were reported other cancers such as melanoma [43]. Our further analysis found PD-L1 positivity did not correlate with the TMB value (Fig. 5c & d), suggesting a complicated and indirect association with gene mutation landscape with PD-1/PD-L1 axis activation in lung cancer. It is known high PD-L1 expression is associated with certain gene mutations including TP53, KRAS, and STK11 [44]. However, the TMB result was assessed by NGS that covers genetic mutation data across the whole genome, including genes that do not necessarily related to immune regulation. Therefore, the correlation between the TMB status and PD-L1 positivity need to be better defined in further study.

The core hypothesis of this study is TMB may act as complementary biomarker with PD-L1 expression to predict patient prognosis. Given most of NSCLC patients enrolled in this study were undergoing targeted therapies, we investigated if combination of TMB with PD-L1 could predict overall survival in of these patients. Survival analysis of ADC subjects showed remarkably longer survival in low/moderate TMB plus negative PD-L1 subgroup compared to other two subgroups (Fig. 6b & c). Several recent clinical trials reported studies in advanced lung cancer showing significant associations between TMB and response to the PD-L1 inhibitor treatment [45, 46]. Here, our data further confirmed TMB in combination with PD-L1 expression could significantly predict patient survival in ADC subjects received platinum-based chemotherapy. One possible reason is that these patients with negative PD-L1 level have developed stronger anti-tumor immunity due to the lack of immunosuppressive molecules while low or moderate gene mutation may associate with dysregulated genes that create immunogenic neoantigens. Of course, given the fact that a lot of other biological mechanisms in addition to TMB and PD-L1 contribute to immunotherapy response, this hypothesis requires more effort and work for further verification. Larger studies are also needed to confirm if TMB is useful for assigning patients single-agent immunotherapies such as checkpoint inhibitors.

In conclusion, our results demonstrated that PD-L1 expression, especially together with TMB status, would serve as an independent predictor of poor prognosis in ADC patients who received targeted treatment. This study is an important step toward understanding the impact of comprehensive genomic profiling in immunotherapy response. Our data showed we should evaluate these two factors independently and maybe use them cooperatively to most accurately define who will benefit from the targeted therapy.

## Conclusions

In summary, we tested the hypothesis that monitoring TMB, in addition to the existing PD-L1 expression level, could represent valuable non-invasive biomarkers for the chemotherapy and targeted therapy. Further analyses are in need to further assess the prognostic value of TMB for NSCLC patients receiving immunotherapy**.**

## Additional file

Additional file 1: **Figure S1**. Comparison of CD8 levels in PD-L1 negative and positive groups. A & B: Comparison of CD8 positive rate in PD-L1 negative and positive groups from ADC (A) and SQCC (B) subjects. C & D: Comparison of CD8 expression levels as distributed by quartiles in PD-L1 negative and positive groups from ADC (C) and SQCC (D). (TIF 427 kb)

## Abbreviations

ADC: adenocarcinoma
IHC: Immunohistochemical
NGS: Next-generation sequencing
NSCLC: non-small cell lung cancer
PCR: polymerase chain reaction
PD-1: programmed death-1
PD-L1: programmed death ligand-1
SQCC: squamous cell carcinoma
TC: tumor cells
TMB: tumor mutational burden
