# Supplementary figures and images for: PD-L1 expression and tumor mutational burden status for prediction of response to chemotherapy and targeted therapy in non-small cell lung cancer
Source: J Exp Clin Cancer Res. 2019 May 14;38:193. doi: 10.1186/s13046-019-1192-1 (PMC6518807; doi:10.1186/s13046-019-1192-1)

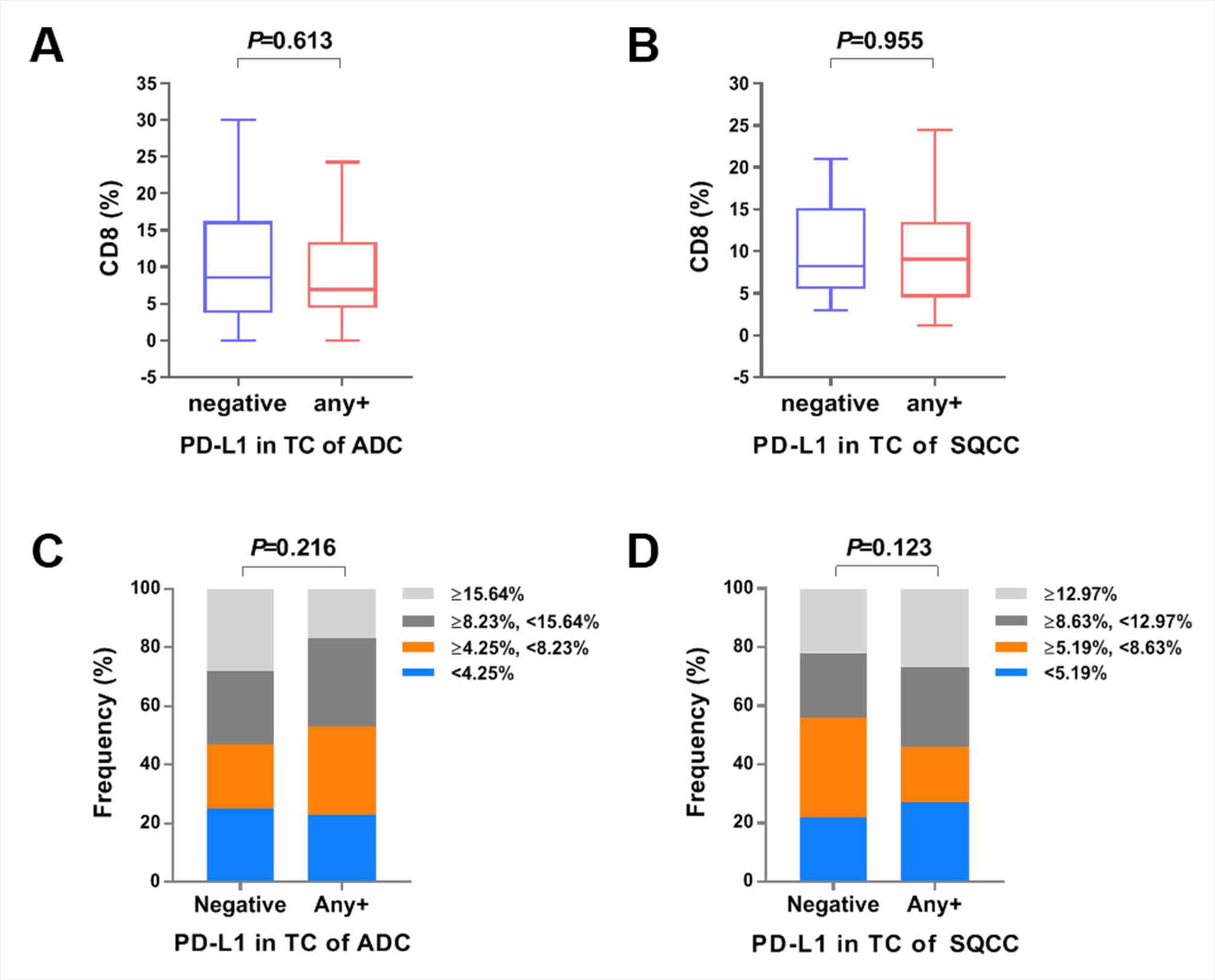

Supplement: Supplementary file 1 — Figure S1. Comparison of CD8 levels in PD-L1 negative and positive groups. A & B: Comparison of CD8 positive rate in PD-L1 negative and positive groups from ADC (A) and SQCC (B) subjects. C & D: Comparison of CD8 expression levels as distributed by quartiles in PD-L1 negative and positive groups from ADC (C) and SQCC (D). (TIF 427 kb) [file 13046_2019_1192_MOESM1_ESM.tif]
